# Nutritional disturbance in acid–base balance and osteoporosis: a hypothesis that disregards the essential homeostatic role of the kidney

**DOI:** 10.1017/S0007114513000962

**Published:** 2013-04-04

**Authors:** Jean-Philippe Bonjour

**Affiliations:** Division of Bone Diseases, Geneva University Hospitals and Faculty of Medicine, Rue Gabrielle-Perret-Gentil, CH-1211Geneva 14, Switzerland

**Keywords:** Nutrition, Osteoporosis, Acid–base balance, Renal proton regulation, Urinary calcium

## Abstract

The nutritional acid load hypothesis of osteoporosis is reviewed from its historical origin to most recent studies with particular attention to the essential but overlooked role of the kidney in acid–base homeostasis. This hypothesis posits that foods associated with an increased urinary acid excretion are deleterious for the skeleton, leading to osteoporosis and enhanced fragility fracture risk. Conversely, foods generating neutral or alkaline urine would favour bone growth and Ca balance, prevent bone loss and reduce osteoporotic fracture risk. This theory currently influences nutrition research, dietary recommendations and the marketing of alkaline salt products or medications meant to optimise bone health and prevent osteoporosis. It stemmed from classic investigations in patients suffering from chronic kidney diseases (CKD) conducted in the 1960s. Accordingly, in CKD, bone mineral mobilisation would serve as a buffer system to acid accumulation. This interpretation was later questioned on both theoretical and experimental grounds. Notwithstanding this questionable role of bone mineral in systemic acid–base equilibrium, not only in CKD but even more in the absence of renal impairment, it is postulated that, in healthy individuals, foods, particularly those containing animal protein, would induce ‘latent’ acidosis and result, in the long run, in osteoporosis. Thus, a questionable interpretation of data from patients with CKD and the subsequent extrapolation to healthy subjects converted a hypothesis into nutritional recommendations for the prevention of osteoporosis. In a historical perspective, the present review dissects out speculation from experimental facts and emphasises the essential role of the renal tubule in systemic acid–base and Ca homeostasis.

‘*It is no exaggeration to say that the composition of the body fluids is determined not by what the mouth takes in but by what the kidneys keep: they are the master chemists of our internal environment. When, among other duties, they excrete the ashes of our body fires, or remove from the blood the infinite variety of foreign substances that are constantly absorbed from our indiscriminate gastrointestinal tracts, these excretory operations are incidental to the major task of keeping our internal environment in an ideal, balanced state.*’

Homer W. Smith (From Fish to Philosopher)^(^ ^1^ ^)^

The hypothesis suggesting that a diet increasing the urinary excretion of acid ion (proton = H^+^) could be a risk factor for osteoporosis was proposed more than 40 years ago^(^ ^2^ ^)^. Conversely, the contention that a diet rich in alkaline or basic (OH^−^) functions would be beneficial to bone health continues to generate a substantial scientific interest. The recurring resurgence of this interest is relayed in the general population by various mass media spreading the belief of small but very active groups of opponents to the use of any animal products^(^ ^3^ ^)^. The same view can be expressed by the scientific community via analyses or meta-analyses of studies which suggest that certain nutrients, particularly animal protein, or foods such as meat or dairy products, by virtue of their supposed ‘acidogenic’ properties, may increase the risk of osteoporosis. At the same time, or in response to these suggestions appearing in the scientific literature, there is growing interest in miraculous benefits claimed for so-called ‘alkalinogenic’ diets or nutritional products, such as those proposed on numerous websites. Consuming ‘alkalis’ will bring about a number of benefits, expanding from hair loss treatment to the prevention of cancers, infections, allergies, obesity, ‘all types of rheumatism’ and, ultimately, osteoporosis, the subject of the present review.

The keen interest in alkali has also found followers among certain anthropologists who argue that the contemporary diet, when compared with that which prevailed before the Neolithic period, has led to osteoporosis together with other diseases linked to the modern way of life, several being hypothetically caused by nutrition-induced metabolic acidosis^(^ ^4^ ^)^.

## Review of acid–base homeostasis

One may be surprised by this keen interest in alkalis and the associated fear of acid, forgetting that the skin or the oesophagus tolerates caustic soda (Na^+^OH^−^) as poorly as hydrochloric acid (H^+^Cl^−^). Yet, basic physiology shows that our bodies are equipped with several systems capable of neutralising or generating protons, such as the bicarbonate–CO_2_ buffer:
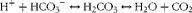



This system enables very effective neutralisation of the excess of H^+^ ions by moving this reaction to the right and therefore increasing the production of CO_2_, which, in physiological conditions, is easily eliminated via the respiratory tract^(^ ^5^ ^)^. In addition to this pulmonary mechanism, the renal tubular system is extremely well equipped to maintain the acid–base balance of the extracellular compartment by modulating the reabsorption of bicarbonate and the secretion of protons. These processes are linked to buffer systems able to eliminate the excess of H^+^ ions produced by cellular metabolism, without substantially lowering urinary pH^(^ ^5^ ^)^. The main urinary buffer systems are:
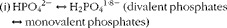


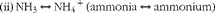



The composition of the extracellular fluid in which the cells of the body exert their specific functions must deviate towards neither the acid nor the alkaline side. Measurable deviations are due to pathological disturbances that affect primarily the digestive tract, intermediary metabolism, the pulmonary system or renal functions. The four classic disturbances of acid–base balance with clinically significant consequences are, on the one hand, acidosis and alkalosis of metabolic origin and, on the other hand, acidosis and alkalosis of respiratory origin^(^ ^5^ ^,^ ^6^ ^)^. Furthermore, deviations from an extracellular pH of 7·35 can be corrected or attenuated by both the capacity of chemical buffers and the physiological regulation at the respiratory and renal tubular levels. The mobilisation of such compensatory mechanisms is expressed by changes in the distribution of buffer system components. These basic concepts are essential for the understanding of the relationship between nutrition and bone health.

As discussed below, the notion of latent acidosis^(^ ^7^ ^)^, as well as the relationship between ageing, renal functional decline and blood acid–base composition^(^ ^8^ ^)^, have been suggested to be causally related to the increased prevalence of osteoporosis in the elderly population. However, alterations in blood pH, [HCO_3_^−^] and/or pCO_2_ have not been documented in relation to changes in the foods or nutrients purported to cause osteoporosis in otherwise healthy individuals^(^ ^9^ ^,^ ^10^ ^)^.

## Is there a reason to question the traditional, accepted approach to analyse acid–base chemistry?

The traditional, accepted bicarbonate-centred formulation of acid–base interpretation was questioned about 25 years ago by Stewart^(^ ^11^ ^)^, who promoted the so-called ‘strong ion difference’ (SID) approach. According to the mathematical model from which this theory was worked out, the components of the volatile bicarbonate–CO_2_ buffer system (CO_2_, HCO_3_^−^, H_2_CO_2_ and CO_3_^2 −^) were dependent variables of the difference in the net charges of fixed cations and anions fully dissociated in solution. Thus, according to Stewart^(^ ^11^ ^,^ ^12^ ^)^, the strong ion difference [Na^+^] − [Cl^−^] or SID would be a determinant of [H^+^]. However, 30 years after Stewart^(^ ^11^ ^,^ ^12^ ^)^, Kurtz *et al.* ^(^ ^13^ ^)^ thoroughly analysed the physico-chemical, physiological and clinical aspects of Stewart's theory when compared with the traditional, accepted bicarbonate-centred approach. In this very comprehensive review^(^ ^13^ ^)^ it was underscored that Stewart's theory^(^ ^11^ ^,^ ^12^ ^)^ reintroduced the confusion in the acid–base literature that existed from the beginning of the twentieth century and had prevailed until the early 1950s. During that period, clinical chemists considered Na^+^ as a base and Cl^−^ as an acid^(^ ^13^ ^)^. Such a consideration entirely disregarded the key position of H^+^ in acid–base reactions. This misconception in clinical acid–base chemistry was dispelled in the mid-late 1950s by Relman^(^ ^14^ ^)^ and Christiensen^(^ ^15^ ^)^, whose ‘prescient analysis foreshadows in some sense the current issues in the literature as they relate to the Stewart framework’^(^ ^13^ ^)^. Furthermore, the bicarbonate-centred approach utilising the Henderson–Hasselbach equation is a mechanistic formulation that reflects the underlying acid–base situation^(^ ^13^ ^)^. It remains the most reliable and used method for physiologists and clinicians to assess acid–base chemistry in human blood^(^ ^13^ ^)^. Therefore, it is inaccurate to claim an absence of consensus as to how to assess acid–base balance by referring primarily to the SID and bicarbonate-centred approaches without emphasising the most cogent arguments developed by Kurtz *et al.* ^(^ ^13^ ^)^. Adherents to the notion of diet-induced acidosis as an essential mechanism for the high prevalence of osteoporosis in the Western world suggest that if no change is observed, this does not mean there is none. However, in order to support the diet-induced acidosis hypothesis of osteoporosis, it would seem necessary to objectively measure whether diet alters blood acid–base equilibrium, and, particularly, whether such alteration can be found in association with bone fragility.

## Nutrition and acid–base balance

In the presence of one of the above-mentioned acid–base balance disturbances, foods, depending on their nutrient composition, can either slightly accentuate or ameliorate a pathological condition. However, in the absence of such pathologies, food components trigger neither extracellular fluid acidosis nor alkalosis.

Any influence of nutritional origin that slightly disrupts the acid–base equilibrium is at once corrected by biochemical buffering systems operating in both the extracellular and intracellular compartments. Then, as indicated above, come into play the homeostatic systems involved in the regulation of pulmonary ventilation and urinary acid excretion via modulation of the renal tubular reabsorption or ‘reclamation’ of filtered bicarbonates and of proton secretion^(^ ^5^ ^)^.

Over the last two decades, tremendous progress has been achieved in understanding the cellular and molecular mechanisms involved in renal tubular acidification (see for reviews Weiner & Hamm^(^ ^16^ ^)^, Hamm *et al.* ^(^ ^17^ ^)^, Koeppen^(^ ^18^ ^)^ and Weiner & Verlander^(^ ^19^ ^)^). Nevertheless, the fundamental concepts elucidated several decades ago on the overall renal control of extracellular proton homeostasis remain valid.

Homeostasis is defined as the stabilisation of the various physiological constants of the ‘internal environment’. It has played an essential part in the evolution of life, from the most elementary unicellular organism to *Homo sapiens*, both in its phylogenetic and ontogenetic trajectories. Bearing in mind the capacity of physiological systems to adapt in response to environmental changes, homeostasis provides a scientific explanation for the basic mechanism of biological evolution^(^ ^1^ ^)^.

Homeostasis includes the maintenance of a constant extracellular concentration of protons. Extracellular levels of other ions such as Na, K, Ca and inorganic phosphate are also barely affected by fluctuations in their respective nutritional intakes, unless their variations are very large in quantity and extend over prolonged periods.

## Claude Bernard's nutrition experiments in rabbits

That diet alters urinary acidity had already been demonstrated in the nineteenth century by Bernard^(^ ^20^ ^)^ in his fundamental experiments on rabbits. By substituting cold boiled beef for their usual dietary regimen (consisting essentially of grass), cloudy, alkaline urine became clear and acidic, like the urine of carnivores^(^ ^20^ ^)^. For this eminent physiologist, whose major contribution was to the elucidation of the homeostasis of the internal environment, these experiments carried out on rabbits represented a particularly cogent example of functional adaptation to environmental variations^(^ ^20^ ^)^. The urinary acidity changes observed in response to food substitution are particularly relevant to the considerations discussed below.

## Origin of the hypothesis involving bone as regulator of acid–base balance

A century after Bernard's^(^ ^20^ ^)^ observations in rabbits, Relman and his colleagues^(^ ^21^ ^–^ ^24^ ^)^ in Boston carried out a series of classical experiments with the objective of establishing, via quantitative data, the vital role of the kidney in acid–base balance. First, in healthy human subjects, i.e. those with normal renal function, Relman *et al.* ^(^ ^21^ ^)^ demonstrated that acid urinary excretion perfectly counterbalanced the net production of non-volatile acid. These experiments showed that the regulation mechanisms for the proton balance were indeed functioning. They signified that, in the absence of renal insufficiency, there was no argument for the involvement of organs other than the kidney in the maintenance of the homeostasis of non-volatile acids.

They then applied their technique to patients suffering from acidosis through chronic renal insufficiency^(^ ^22^ ^,^ ^24^ ^)^. In these studies, carried out on a small number of patients with a pathologically decreased but stable serum level of bicarbonates, their method of calculation indicated a positive balance of protons^(^ ^22^ ^,^ ^24^ ^)^. This led to the hypothesis that the quantity of acid retained in the body, indirectly estimated and not measured, was neutralised by the release of bicarbonates by the dissolution of bone mineral^(^ ^22^ ^,^ ^24^ ^)^. (Bone mineral is not pure hydroxyapatite. The apatite crystals contain impurities, most notably carbonate (CO_3_^2 −^) in place of the phosphate group. The concentration of carbonate (4–6 %) makes bone mineral similar to a carbonate apatite. Other documented substitutions are K, Mg, Sr and Na in place of the Ca ions, and Cl and F in place of the hydroxyl groups. These impurities reduce the crystallinity and solubility of the apatite^(^ ^25^ ^)^.)

In order to document this hypothetical bone mobilisation of bicarbonates, the Relman team carried out an initial study on five normal subjects^(^ ^23^ ^)^. The administration of large doses of NH_4_Cl, drastically decreasing the blood level of bicarbonates from 26·5 to 18·8 mEq/l, was associated with a negative Ca balance, attributed to the mobilisation of calcium carbonate of skeletal origin^(^ ^23^ ^)^. This interpretation was therefore based on the measurement of a decreased but stable level of bicarbonates, whereas during the same period, the estimate of acid balance indicated a progressive accumulation of protons^(^ ^23^ ^)^. The negative Ca balance was due to an increase in urinary losses, the intestinal Ca absorption being unchanged^(^ ^23^ ^)^. The change in the rate of urinary Ca excretion was therefore interpreted as a consequence of the mobilisation of Ca from the bones, associated with the release of buffer substances due to the dissolution of bone mineral in the presence of severe metabolic acidosis^(^ ^23^ ^)^. The authors did not consider the possibility that the mobilisation of bone Ca might be secondary to an effect on the renal tubular reabsorption of Ca. In several subsequent studies, it turned out that acidosis is a factor that considerably inhibits the tubular reabsorption of Ca^(^ ^26^ ^)^. Consequently, the mobilisation of bone Ca observed in these earlier experiments^(^ ^23^ ^)^ may therefore actually represent a secondary phenomenon, compensating for the tendency towards hypocalcaemia rather than being the cause of the negative Ca balance^(^ ^26^ ^)^.

In a subsequent study from the same group, Ca balance was determined in eight patients suffering from severe renal insufficiency^(^ ^24^ ^)^. In the majority of these patients, there were signs of osteodystrophy including generalised skeletal demineralisation and radiological evidence of secondary hyperparathyroidism as expressed at the phalanges by the presence of sub-periosteal resorption^(^ ^24^ ^)^. Administration of NaHCO_3_, causing an increase in the concentration of serum bicarbonates from 18·7 to 27·4 mEq/l and thereby correcting metabolic acidosis, was associated with modest improvement in the negative Ca balance, from − 5·3 to − 1·5 mEq/d. Moreover, this correction was essentially due to a decrease in the faecal excretion of Ca, the urinary excretion being considerably reduced in these patients^(^ ^24^ ^)^.

## From severe renal metabolic acidosis to the hypothesis of ‘latent’ acidosis of nutritional origin

The three above-mentioned studies, two conducted in patients with chronic renal insufficiency^(^ ^22^ ^,^ ^24^ ^)^ and one in normal subjects rendered severely acidotic through the administration of NH_4_Cl^(^ ^23^ ^)^, are the basis of the hypothesis that bone mineral plays an important part in whole-body acid–base balance. This role would rely on the mobilisation of alkaline ions from the bone, thereby offsetting the excess of acid. This hypothesis is still being considered as a well-established scientific fact. The putative bone buffer mobilisation would be operational not only in the case of severe renal insufficiency, but also in the absence of any pathology, affecting the respiratory and/or renal regulatory systems involved in the maintenance of acid–base balance.

According to this hypothesis, the ‘Western diet’, in particular, would be a risk factor for osteoporosis, as it may supply an excess of protons that the pulmonary and renal systems would no longer be in a position to eliminate and which, therefore, would require the mobilisation of calcium bicarbonate from the bone tissue. However, it has been demonstrated that, in subjects in good health, blood pH and serum level of bicarbonates are not altered following dietary manipulations that induce alterations in urinary proton excretion, such as quantitative variations in the protein intake or qualitative differences in the diet, when comparing omnivorous and vegetarian subjects^(^ ^27^ ^–^ ^29^ ^)^. In the absence of studies demonstrating the existence of an acid–base imbalance in the extracellular fluid, the notion of a ‘latent’ metabolic acidosis state has been put forward^(^ ^7^ ^)^. This expression appears to be a misuse of language. The term ‘latent’ in a medical context is used to describe a state during which a clearly identified pathological disturbance or a pathogenic agent of a disease is detectable but remains inactive. A good example is the varicella zoster virus that remains latent after the initial bout of chicken pox has ended. When the virus becomes reactivated, usually several decades later, it causes herpes zoster. However, this phenomenon does not apply to the putative relationship between metabolic acidosis, the incriminated state of nutritional origin and osteoporosis. Therefore, the systemic acidosis of pure dietary origin remains a hypothesis that has not been scientifically demonstrated but which, in a certain number of publications (see below), is considered to be a proven pathophysiological mechanism leading to osteoporosis.

Even the hypothesis that bone is very important in maintaining stable serum HCO_3_^−^ in established chronic metabolic acidosis has been challenged on the grounds of both theory and experimental data^(^ ^30^ ^–^ ^32^ ^)^.

## Bone alkali store and overestimated proton retention in chronic renal failure

Even if one admits that in the experiments conducted in patients suffering from acidosis due to chronic renal insufficiency^(^ ^22^ ^,^ ^24^ ^)^, the stability of low serum bicarbonates would be the consequence of some alkali mobilisation from an endogenous source, the origin cannot be the bone mineral^(^ ^30^ ^–^ ^32^ ^)^. Indeed, the quantity of buffering substances released from the bone would be largely insufficient to neutralise the acid assumed to have accumulated in the course of years when chronic renal insufficiency has been developing^(^ ^30^ ^–^ ^32^ ^)^. It was estimated that about 50 % of bone mineral would have to be dissolved over approximately 1·8 years in order to achieve such an acid neutralisation^(^ ^30^ ^–^ ^32^ ^)^. In other words, calculation based on the total Ca and alkali content in the skeleton indicates that with a supposed proton retention of 12–19 mEq daily in chronic renal acidosis^(^ ^22^ ^,^ ^24^ ^)^, it would take 3·6 years for the bone alkali store to be exhausted in order to buffer this amount of acid^(^ ^32^ ^)^.

Thus, a quantitative estimate of the bone alkali content rules out that mobilisation of apatite mineral would be implicated in the maintenance of the low serum level of bicarbonates observed in the metabolic acidosis of chronic renal insufficiency.

A re-evaluation of the various components of acid–base balance^(^ ^32^ ^,^ ^33^ ^)^ made highly questionable the hypothesis that bone alkali mobilisation is an important process in maintaining a stable low level of serum bicarbonate in chronic metabolic acidosis^(^ ^30^ ^–^ ^32^ ^)^. Important technical progress has made possible the determination of the net gastrointestinal absorption of alkali, applying a method that avoids imprecise measurements of the quantities consumed and excreted in the faeces^(^ ^30^ ^–^ ^32^ ^)^. With the use of this technique, as well as taking into account the urinary excretion of organic cations and anions (see below), the acid–base balance appeared to be neutral in end-stage renal disease patients^(^ ^33^ ^,^ ^34^ ^)^. Consequently, with no excess of protons to be neutralised, there was no reason to invoke the mobilisation of alkali from the bone tissue in chronic renal insufficiency with stable metabolic acidosis.

Thus, a technical error, corresponding either to an underestimate of the net quantity of acid excreted, or to an overestimate of the net acid production, has perpetuated the incorrect concept that bone mineral plays a substantial part in acid–base balance in patients suffering from chronic renal acidosis. This incorrect concept does not mean that the acidosis generated by severe chronic kidney disease would not contribute to renal osteodystrophy. Nevertheless, other mechanisms probably play a more important part than acidosis *per se* in the deterioration of bone integrity in the case of severe chronic renal failure (for a review, see Hruska & Mathew^(^ ^35^ ^)^).

## Extrapolation from severe metabolic acidosis in rodents to the putative nutritional protein origin of osteoporosis in the general human population

The effects of metabolic acidosis on the skeleton were examined both *in vitro* and *in vivo* in animal experiments^(^ ^36^ ^–^ ^40^ ^)^. The results of these studies have been interpreted as supporting the hypothesis of an acid-buffering role of bone mineral. They are considered as experimental evidence in favour of the putative causal relationship between the so-called ‘Western diet’ and the prevalence of osteoporosis in the general population^(^ ^4^ ^,^ ^7^ ^,^ ^41^ ^–^ ^44^ ^)^.

Furthermore, these observations, whether on isolated bone cells or on rodents^(^ ^36^ ^,^ ^37^ ^,^ ^38^ ^–^ ^40^ ^)^, taken together with the fact that food intake modifies the degree of acidification of urine, as already demonstrated by Bernard^(^ ^20^ ^)^ in the mid-nineteenth century, provided the rationale for exploring whether there would be a possible relationship between protein intake and osteoporosis, and, particularly, whether protein from animal *v.* vegetable sources would be more detrimental to bone health. To this end, many epidemiological studies have been published in the course of the last 16 years^(^ ^45^ ^–^ ^56^ ^)^. Several of these reports appear to present some methodological flaws. Examples include the following: the age of the included subjects (varying between 35 and 74 years); the absence of an analytical distinction between sex; the inclusion of both pre-menopausal and postmenopausal women; the scarce or rather poor estimation of physical activity; the non-appreciation of the risk of falls; the variable levels of protein intake, often with average consumption above the recommended nutritional intake, therefore limiting the impact of protein malnutrition. In such disparate clinical conditions, it seems questionable to draw a synthesis from these studies by calculating an average relative risk with regard to the development of low bone mineral density (BMD)/content and/or fracture risk.

Furthermore, in some reports testing the *a priori* hypothesis that acidic urinary excretion (particularly when positively related to protein intakes) would reflect metabolic acidosis and thereby should be associated with poor bone health, the data were *a posteriori* equivocally handled in favour of the postulated assumption. Thus, when the whole cohort did not show any associated relationship, further analysis focused on subgroups as computed by cross-tabulation combining highest protein with lowest Ca intakes^(^ ^53^ ^)^, or on subjects with a history of fracture exclusively^(^ ^54^ ^)^, or still on participants with high, but not with low urinary acid excretion^(^ ^57^ ^)^.

## Evaluation of the acid and alkali nutritional load

Starting from the hypothesis that the quantity of residual acid in the diet would influence the bone integrity of subjects otherwise in good health, several methods were proposed, based on studies conducted in the context of chronic renal insufficiency. First, it should be specified that measuring the pH of foods does not reflect the acid or alkali load they provide to the body. For example, orange juice has a low pH, by virtue of its high citric acid content, whereas once it has been ingested, it adds an alkali load to the body. Sulphurous amino acids (R-S) are neutral, but add acid loads once they have been metabolised, the reaction being:
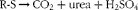



Foods contain numerous chemical substances. Their absorption depends not only on the type of substances ingested, but also on interactions with gastric acid and other nutrients in simultaneously ingested foods. Therefore, it is almost impossible to predict the impact of food ingestion on the regulation of acid–base balance^(^ ^32^ ^)^.

Moreover, since the intestinal absorption of the acid or alkali loads of food is incomplete, it is still necessary to be able to measure their quantity when excreted in the faeces. Taking into account both the experimental and analytical difficulties associated with such measurements, a simplified method has been developed and validated among subjects with chronic renal acidosis^(^ ^31^ ^,^ ^32^ ^–^ ^34^ ^,^ ^58^ ^)^. According to this method, in the steady state, the total amount of inorganic cations 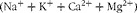 minus the total amount of anions 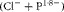 measured in the urine over 24 h can be used to estimate the net gastrointestinal absorption of alkalis. This measurement has the advantage of also including any other source of alkalis translocated into the extracellular environment, hypothetically including those from the bone tissue^(^ ^31^ ^)^.

## Mathematical model to estimate the potential renal acid load of foods

The principle according to which, at the steady state, the quantity of electrolytes excreted in the urine equals their quantity absorbed by the intestine has led to the development of mathematical models in order to estimate the relationship between food intake and net renal acid excretion (NAE)^(^ ^59^ ^)^. NAE includes the daily urinary excretion of both inorganic and organic acids. This measurement provides an estimate of net endogenous acid production (NEAP)^(^ ^60^ ^)^. The analytical difficulty relating to the measurement of urinary organic acids (OA), which include citric, lactic, oxalic, malic and succinic acids, as well as glutamic and aspartic amino acids, has been circumvented by an estimate derived from the body surface. The equation used is:
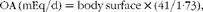
in which the value 41 corresponds to the median daily urinary excretion of OA for an average body surface of 1·73 m^2^ among subjects in good health^(^ ^60^ ^,^ ^61^ ^)^. This anthropometric estimate of OA is included in the calculation of the potential renal acid load (PRAL) of foods^(^ ^62^ ^)^.

This calculation avoids the direct measurement of NAE, which is already an indirect measurement in itself of the NEAP. The PRAL can be estimated relatively easily from dietary studies, using weekly diaries or regular questionnaires, in which the quantities ingested are analysed according to nutritional composition tables. The nutrients taken into account for the PRAL calculation are: (phosphorus+protein) − (K+Ca+Mg).

The estimate of endogenous acid production has been further simplified by considering only protein and K intakes^(^ ^63^ ^)^. An analysis of about twenty different diets followed by 141 subjects aged 17–73 years showed a coefficient of correlation (*R* ^2^) of 0·36 (*P*= 0·006) with a positive slope between protein intake and renal net acid excretion (RNAE, taken as a NEAP index), whereas it was 0·14, with a negative slope, for K intake^(^ ^63^ ^)^. By the regression of the protein:K ratio, the *R* ^2^ became 0·72 (*P*< 0·001)^(^ ^63^ ^)^. The use of this simple ratio estimates the acid load of foods according to the following equation^(^ ^63^ ^)^:
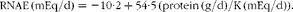



Physiologically, the meaning of the protein:K ratio remains obscure. Indeed, K *per se* cannot be considered as an alkalinising ion, since hyperkalaemic states are usually the generator of acidosis and not of metabolic alkalosis^(^ ^6^ ^)^.

Of note, the development of a tool enabling the estimation of the PRAL of foods was aimed at modifying the urinary pH by dietetic means, particularly in the context of preventing recurrent urinary lithiasis^(^ ^62^ ^)^. Thus, taking into account the differences in pH-dependent mineral solubility, the nutritional approach for preventing the recurrence of calcium phosphate or uric acid lithiasis, for example, has consisted in promoting acidification or alkalinisation of urine, respectively (see for a review Grases *et al.* ^(^ ^64^ ^)^ and Moe *et al.* ^(^ ^65^ ^)^).

## Relationship between bone health and the acid or alkaline load of the diet

Over the last two decades, several reports have considered the relationship between the Ca economy and bone metabolism and K intake from foods or from the administration of potassium bicarbonate or citrate salts^(^ ^42^ ^,^ ^66^ ^–^ ^75^ ^)^. In the context of osteoporosis, human intervention studies have been designed to test whether the administration of alkalinising salts may favourably affect Ca and bone metabolism and therefore eventually be developed as anti-osteoporotic therapy^(^ ^66^ ^,^ ^67^ ^,^ ^69^ ^–^ ^75^ ^)^. The results obtained by the end of relatively short time interventions suggested that taking alkalinising salts may transiently reduce bone turnover markers, and/or increase the balance of bone health, and thus lead to ‘…tipping the scales in favour of potassium-rich, bicarbonate-rich foods’^(^ ^42^ ^)^. However, prolonged randomised studies did not confirm such a positive influence on Ca economy and bone loss prevention^(^ ^72^ ^,^ ^73^ ^)^. Decreased intestinal Ca absorption can explain reduced calciuria (UCa), with K salts yielding no significant net change in Ca balance^(^ ^70^ ^,^ ^73^ ^)^. Furthermore, in terms of skeletal health, in a 2-year randomised placebo-controlled trial in healthy postmenopausal women aged 55–65 years, potassium citrate administered in two doses (moderate: 18·5 mEq/d and high: 55·5 mEq/d) had no persistent effect on biochemical markers of bone remodelling measured at regular intervals. In line with this negative assessment, the reduction in areal BMD observed at the end of the intervention did not slow down, despite an increase in urinary pH and excretion of K in the course of 2 years of treatment^(^ ^72^ ^)^. In this trial, the consumption of additional fruits and vegetables (+300 g/d) increasing the urinary excretion of K neither reduced bone turnover nor prevented areal BMD decline when compared with the placebo group^(^ ^72^ ^)^. As reported in short-term studies, a temporary reduction in bone markers was observed 4–6 weeks after the start of the treatment^(^ ^72^ ^)^. In other words, the classical study supporting the ‘benefits’ of nutritional alkalinisation for bone health^(^ ^66^ ^)^ was not confirmed by a long-term clinical trial, not only measuring bone remodelling, but also bone loss following the menopause, at two skeletal sites of extreme importance in the risk of osteoporotic fractures – spine and proximal femur^(^ ^72^ ^)^.

Despite this negative evidence from a well-designed clinical trial^(^ ^72^ ^)^ and long-term preclinical investigations showing no relationship between urinary acid excretion and either bone status (density and strength) or remodelling^(^ ^76^ ^)^, the idea that taking bicarbonates or alkaline K salts would be beneficial to the Ca economy and might result in better bone health and thereby prevent osteoporotic fractures continues to generate reports aimed at demonstrating such a therapeutical possibility^(^ ^43^ ^,^ ^57^ ^,^ ^74^ ^,^ ^77^ ^)^.

In the context of osteoporosis prevention in postmenopausal women and the elderly, modification of dietary habits could be plausible so long as long-term efficacy can be clearly demonstrated. In contrast, the daily consumption of alkaline salt preparations over several decades appears to be hazardous in the absence of an evaluation of possible long-term toxicity. For example, the risk of enhancing vascular calcifications cannot be ruled out, particularly when alkaline salts are combined with Ca and vitamin D supplementation. In the study by Jehle *et al.* ^(^ ^71^ ^)^, the lumbar spine BMD difference without a consistent change in bone remodelling markers between the potassium citrate and potassium chloride groups could, as suggested by the authors, be fully attributed to the enhanced non-cellular matrix mineralisation and thus be largely independent of bone cell-mediated events. Whether such Ca deposition in soft tissues, resulting from the consumption of alkaline salt supplements and an increased supply of Ca–vitamin D, could also occur in the cardiovascular system^(^ ^78^ ^,^ ^79^ ^)^ is unclear and is a risk that could overbalance the small and inconsistent benefit over placebo observed on bone integrity with alkaline supplements after 1 or 2 years of intervention^(^ ^71^ ^,^ ^72^ ^,^ ^80^ ^)^.

## Reviews of studies dealing with the dietary acid load hypothesis of osteoporosis

Recent reports have not sustained the existence of a pathophysiological mechanism linking the consumption of some nutrients, particularly animal protein, to the induction of a biologically significant metabolic acidosis that would result in a negative Ca balance, bone loss and eventually osteoporotic fracture.

A first meta-analysis including twenty-five clinical trials, and adhering to rigorous pre-defined quality criteria, focused on the association between NAE and UCa^(^ ^81^ ^)^. The analysed trials consisted in nutritional treatment and were carried out on healthy subjects in order to test the effect of either two types of food (meat *v.* soya), or certain nutrients (quantity of protein or dairy protein *v.* soya protein), or even acidifying (NH_4_Cl) or alkalinising (citrate, sodium bicarbonate or K) salt supplements. A significant linear relationship was found between net acid excretion and Ca excretion for both acidic and alkaline urine^(^ ^81^ ^)^. Whether this increase in UCa when associated with net acid excretion would correspond to a decrease in Ca balance was examined in another meta-analysis^(^ ^82^ ^)^. The included studies had all employed stringent methods to measure Ca balance and bone metabolism in relation to changes in NAE^(^ ^82^ ^)^. The treatments were carried out on adult subjects in good health and consisted of modifications of protein intake, in terms of quantity or quality^(^ ^82^ ^)^. Despite an increase in UCa in response to the nutritional treatment, Ca balance, as well as bone resorption evaluated by measuring the type I collagen N-telopeptide, did not show any correlation with the acid load of the dietary regimens tested^(^ ^82^ ^)^. This meta-analysis did not suggest that protein-induced UCa associated with increased NAE would exert a negative impact on bone health, leading to osteoporosis in the long term. Therefore, it does not argue in favour of the theory advocating alkaline diets.

Furthermore, two other recent original reports did not sustain the hypothesis that a high dietary acid load might be detrimental to bone integrity. In the Framingham Osteoporosis Study, dietary acid load, estimated by the NEAP and PRAL, was not associated with BMD at any skeletal sites among 1069 ‘Original’ and 2919 ‘Offspring’ cohort participants^(^ ^83^ ^)^. A possible exception was in older men with a trend between the NEAP and the femoral neck but not lumbar spine BMD, whereas no association was found with PRAL^(^ ^83^ ^)^. Moreover, there was no interaction between either the NEAP or PRAL and total Ca intake^(^ ^83^ ^)^. Thus, this study did not support the hypothesis that a high dietary acid load combined with a relatively low Ca intake might accelerate bone loss and increase the risk of fragility fracture^(^ ^83^ ^)^. Another report was quite consistent with the detailed analysis of the data from the two Framingham generation cohorts^(^ ^83^ ^)^. Indeed, no apparent relationship was found between urinary pH or urinary acid excretion and either the change in lumbar or femoral BMD or in the incidence of fractures after 5 years of monitoring including approximately 6800 person-years (age at baseline: approximately 59 years; female sex: 70 %) in a prospective investigation^(^ ^9^ ^)^.

Another recent and comprehensive review reported on a systematic search of the published literature for randomised intervention trials, prospective cohort studies and meta-analysis of the acid-ash or acid–base hypothesis in relation to bone-related outcomes. In these studies, the dietary acid load was altered, or an alkaline diet or alkaline salts were provided to healthy human adults^(^ ^10^ ^)^. The objective of this systematic review was to evaluate the relationship between the dietary acid load and osteoporosis using Hill's epidemiological criteria of causality^(^ ^84^ ^)^. It was concluded that a causal association between the dietary acid load and osteoporotic bone disease is not supported by evidence, nor that an alkaline diet favourably influences bone health^(^ ^10^ ^)^. Furthermore, assuming that fruit and vegetables are beneficial to bone health, such a positive influence would be mediated by mechanisms other than those related to their alkalinising potential, as experimentally demonstrated several years ago^(^ ^85^ ^)^.

## Randomised clinical trials with potassium alkali in postmenopausal women

The bone data from two independent long-term randomised clinical trials testing K alkali supplements against placebo in healthy postmenopausal women^(^ ^69^ ^,^ ^72^ ^)^ have been analysed in one single publication^(^ ^80^ ^)^. This analysis clearly shows, after 2 years of intervention, that K alkali treatment does not alter BMD changes at both lumbar spine and hip levels and has no effect on markers of bone resorption^(^ ^80^ ^)^. Therefore, the previously reported long-term persistence of the urine Ca-lowering effect of potassium bicarbonate^(^ ^69^ ^)^ was not associated with a significant benefit in terms of postmenopausal osteoporosis prevention^(^ ^80^ ^)^. Likewise, both the greater spinal or hip BMD and the lower bone resorption markers, which were found to be associated with reduced estimates of NEAP and higher dietary K intakes in cross-sectional population studies of pre- and postmenopausal women^(^ ^86^ ^,^ ^87^ ^)^, were not confirmed in long-term randomised trials^(^ ^72^ ^,^ ^80^ ^)^. When compared with the null finding of these two trials^(^ ^72^ ^,^ ^80^ ^)^, a report, still in press^(^ ^77^ ^)^, describes a positive effect of potassium citrate associated with supplements of calcium carbonate and vitamin D_3_ on BMD. This effect, recorded in a 2-year randomised trial carried out in healthy, elderly men and women studied together, remains to be mechanistically explained since it was observed, as in the two above-mentioned studies^(^ ^72^ ^,^ ^80^ ^)^, in the absence of any persistent reduction in bone resorption markers^(^ ^77^ ^)^.

## Phosphate intake and calcium balance

The dietary acid load hypothesis also postulates that increasing the urinary excretion of phosphate, considered as an ‘acidic’ ion, enhances UCa and contributes to the loss and fragility of bones with ageing^(^ ^59^ ^,^ ^88^ ^,^ ^89^ ^)^. In sharp contrast with this hypothesis but in full agreement with physiological notions on the phosphate–Ca interaction^(^ ^90^ ^)^, analysis of twelve human studies indicated that higher phosphate intakes were associated with decreased UCa and improved Ca balance^(^ ^91^ ^)^.

## Age decline in renal function and osteoporosis: are they causally related?

It can be argued that the age-related decline in renal function, with its associated trend towards metabolic acidosis, would be sufficiently important to accelerate bone resorption while reducing bone formation^(^ ^8^ ^)^, and thus could eventually explain the increased incidence of osteoporotic fractures with ageing. According to this putative pathophysiological mechanism, it would be justified to treat age-related osteoporosis by potassium bicarbonate administration or by appropriate modifications of the net dietary acid–base load^(^ ^8^ ^,^ ^66^ ^)^. However, there is no evidence that elderly patients with established osteoporosis, as documented by either spine or hip BMD *T*-score ≤ − 2·5 or by one prevalent vertebral fracture, have a lower glomerular filtration rate and more severe metabolic acidosis^(^ ^92^ ^)^ compared with age- and sex-matched non-osteoporotic subjects^(^ ^93^ ^,^ ^94^ ^)^. Furthermore, in the National Health and Nutrition Examination Survey (NHANES) III population, a much larger number of subjects have osteoporosis/osteopenia^(^ ^95^ ^)^ rather than a low glomerular filtration rate^(^ ^93^ ^)^ or metabolic acidosis^(^ ^94^ ^)^. In the analysis of the NHANES III survey, BMD was not found to be diminished by mild or moderate renal insufficiency^(^ ^96^ ^)^. In fact, renal function itself was not independently associated with BMD, after taking into account sex, age and body weight^(^ ^96^ ^)^. Furthermore, in this large survey, changes in serum bicarbonate were not apparent until chronic renal insufficiency, as estimated by the Cockcroft–Gault creatinine clearance, was ≤ 20 ml/min^(^ ^97^ ^)^. Taken together, these results do not support the notion that age-related metabolic acidosis that would result from the deterioration of renal function could be pathophysiologically implicated in the marked increase in the prevalence of osteoporosis observed with ageing in the general population.

## Conclusions

It is a well-established biological fact that the degree of urinary acidity varies according to the type of consumed foods. In the middle of the nineteenth century, Bernard^(^ ^20^ ^)^ considered this variation to be an example of physiological control in the internal environment. A century later, experiments carried out among patients suffering from severe metabolic acidosis caused by renal insufficiency, or among healthy subjects made acidotic by administering NH_4_Cl, suggested the involvement of bone tissue in maintaining the acid–base balance. This hypothesis was later refuted on the basis of both theoretical and experimental arguments. Despite this rebuttal, the hypothesis was put forward that bone could play a buffering role, with the consideration that nutrients, particularly animal proteins with their acid load, could be a major cause of osteoporosis. Several recent human studies have shown that there is no relationship between nutritionally induced variations of urinary acid excretion and Ca balance, bone metabolism and the risk of osteoporotic fractures. Variations in human diets across a plausible range of intakes have been shown to have no effect on blood pH. Consistent with this lack of a mechanistic basis, long-term studies of alkalinising diets have shown no effect on the age-related change in bone fragility. Consequently, advocating the consumption of alkalinising foods or supplements and/or removing animal protein from the human diet is not justified by the evidence accumulated over the last several decades.
